# A Happy New-Year

**Published:** 1856-01

**Authors:** 


### EDITORIAL DEPARTMENT.

---

Will soon be published, from the press of Messrs. Blanchard & Lea, of Philadelphia, "The Principles and Practice of Physical Exploration, as applied to the Diagnosis of Diseases of the Respiratory Organs. By Austin Flint, M. D."

*Octavo of from* 400 *to* 500 *pages.*

The well known ability of the writer is a sufficient guarantee that this work will be a valuable contribution to that department of medical research. We hope that it may meet with a warm reception among the author's old friends, the subscribers of the Buffalo Medical Journal.

---

*A Happy New-Year.*—To all the known and unknown friends for whom we labor every month, we send this our New-Year's Greeting. Few can appreciate the nearness and cordiality of the feeling which an editor has for his readers. With all of them he has a *quasi* acquaintance, in their constant intercourse he comes to regard them all as familiar friends, and it is no cold formality with us, when we utter the good old wish for blessing—a Happy New-Year!

For the year past some sad regrets may dim the gayety of the present time. The thought of the dead comes up; of Bartlett—brightest of the stars extinguished—and of the "noble army of martyrs" at Norfolk and Portsmouth. The winds of winter howl above the sepulchres of none more gallant, generous, and Godlike than these! They sleep to know no waking till they hear the life-giving words, "I was sick and ye comforted me!"

For the year to come let the word be Progress! In our short lives there is too little time for labor, to waste it in backward glances at the past. Working earnestly and sincerely, and living singly to our profession, we shall earn a claim to another Happy New-Year in 1857, by our fidelity to our mission in 1856.

